# Juvenile Wels Catfish (*Silurus glanis*) Display Age-Related Mortality to European Catfish Virus (ECV) under Experimental Conditions

**DOI:** 10.3390/v14081832

**Published:** 2022-08-21

**Authors:** Flóra Abonyi, Ádám Varga, Boglárka Sellyei, Edit Eszterbauer, Andor Doszpoly

**Affiliations:** Veterinary Medical Research Institute, 21 Hungária krt., H-1143 Budapest, Hungary

**Keywords:** European catfish virus, ranavirus, wels catfish, virus challenge, age-related mortality

## Abstract

We have limited knowledge about the course of the European catfish virus (ECV) infection in different age groups of wels catfish (*Silurus glanis*). The results of this study demonstrate that an ECV strain isolated from the brown bullhead (*Ameiurus nebulosus*) in Hungary could cause devastating losses among juvenile wels catfish. Furthermore, the age-related mortality rate following ECV infection was investigated in three virus challenge experiments at two different virus dosages. Eight-week-old (ca. 3 g), ten-week-old (ca. 8 g), and sixteen-week-old (ca. 55 g) catfish were infected with ECV at 21°C. In the youngest age group, 96% (at a 10^6^ TCID_50_/mL dosage) and 100% (at 10^5^ TCID_50_/mL) mortality rates were observed, while these rates were reduced to 56% and 68% in the ten-week-old groups, respectively. The mortality was significantly higher in the virus-exposed groups than in the control ones. In the sixteen-week-old group, 23% mortality was detected at a 10^5^ TCID_50_/mL concentration of ECV. Here, a significant difference was not found between the exposed and control groups. The performed experiments show that different age groups of wels catfish may have various susceptibility to ECV. These findings draw attention to the importance of the prevention of/protection against virus infections in juvenile (up to 3-month-old) wels catfish in aquaculture.

## 1. Introduction

Aquaculture is one of the most dynamically developing agricultural sectors worldwide. Viruses causing high morbidity and mortality are one of the biggest problems in veterinary health management both in the wild and in captive fish populations [1]. The family *Iridoviridae* is composed of seven genera: *Chloriridovirus*, *Daphniairidovirus*, *Decapodiridovirus*, *Iridovirus*, *Lymphocystivirus*, *Megalocystivirus*, and *Ranavirus*. The genus *Ranavirus* currently contains seven species recognized by the International Committee on Taxonomy of Viruses: *Ambystoma tigrinum virus*, *Common midwife toad virus*, *Epizootic hematopoietic necrosis virus*, *European North Atlantic ranavirus*, *Frog virus 3*, *Santee-Cooper ranavirus*, and *Singapore grouper iridovirus*. Ranaviruses (RVs) belonging to the genus *Ranavirus* (family *Iridoviridae*) have large, double-stranded DNA genomes, 100 to 140 kilobase pairs in length [2,3,4]. RVs cause acute, systemic diseases and are often responsible for mass mortality, as reported from many countries worldwide [5]. They have been recovered from fish, amphibian, and reptilian species [6,7]. RVs were discovered in amphibians in the mid-1960s [8]. In fish, the Epizootic hematopoietic necrosis virus (EHNV) was the first RV to be isolated from a mass mortality event in 1986 in Australia [9]. Later, two morphologically and genetically similar viruses, the European sheatfish virus (ESV) and the European catfish virus (ECV) were assigned to the species. ECV outbreaks were detected in black and brown bullheads (*Ameiurus melas* and *A. nebulosus*) in France [10,11], in Italy [12], and in Hungary [13], while ESV was isolated from wels catfish (*Silurus glanis*) in Germany [14] and in Poland [15]. Furthermore, it was reported in turbot (*Scopthalmus maximus*) in Denmark [16] and in pike-perch (*Sander lucioperca*) in Finland [17].

In Hungary, the first RV outbreak was reported in 2008. Mass mortality of the brown bullhead (*Ameiurus nebulosus*) was observed, and the causative agent, ECV, was successfully isolated (Juhász et al. 2013). The next mass mortality events were documented during the spring of 2012. Diseased and dead brown bullheads were collected from two separated populations, and the gross pathological examination recorded hemorrhages in the skin, fins, gills, and internal organs (including the liver and kidney) [13]. The whole-genome sequence of the latter two RV isolates confirmed that they were very closely related to the formerly described ESV isolate [18].

The Wels catfish has come to be an important freshwater fish species, increasingly being involved in intensive farming in Europe and also in Hungary [19]. Besides their economic importance, they have huge ecological significance in wild fish populations.

Although the susceptibility of wels catfish to different species of RVs has been investigated [20], up to now, the role of the age of the fish as a predisposing factor has not been clarified in regard to the mortality caused by the ECV. Therefore, in this study, we experimentally examined the susceptibility of wels catfish fingerlings of different ages to ECV, and we demonstrated that the Hungarian ECV isolate from the brown bullhead could cause severe decline in wels catfish populations.

## 2. Materials and Methods

### 2.1. Ethics Statement

All animal experiments were performed under a Hungarian National Scientific Ethical Committee on Animal Experimentation license (PE/EA/403-7/2019). The minimum number of fish required to obtain statistically reliable results was used. All efforts were made to minimize suffering.

### 2.2. Animals

Animal experiments were performed using wels catfish fingerlings from a Hungarian fish farm where ECV or other RVs were not detected. The fingerlings were raised on a fish farm in large tanks supplied with thermal water (26 °C) (used in Experiments (A) and (B)). Later, the fish were moved and kept in the animal facility of the University of Debrecen in a recirculating aquaculture system (24 °C) (used in Experiment (C)). The fish were transported to the Veterinary Medical Research Institute’s animal facility two weeks prior to the experiments and were kept at 24–26 °C in a flow-through tank system, and fed with commercial pelleted food (BioMar EFICO Alpha, Brande, Denmark) once a day. Before Experiments (B) and (C), FMC (formalin, malachite green, methylene blue) treatment at 0.6–0.75 mL/50 L concentration was conducted for three consecutive days to prevent the proliferation of ectoparasites.

### 2.3. Virological, Bacteriological, and Parasitological Examinations

During the acclimatization period, fish selected randomly were tested for the presence of RVs by PCR [21], and scraping samples were also taken from the skin and gills for parasitological screening using light microscopy. 

For bacteriological examinations, the spleen and kidney of dissected fish of Experiment C were sampled with sterile inoculating loops. Bacterium colonies with alpha- or beta-haemolytic activity were isolated on Columbia agar, supplemented with 5% defibrinated sheep blood (Biolab Zrt., Budapest, Hungary). Cytochrome oxidase activity was detected using Microbiology Bactident Oxidase strips (Merck, Germany). To identify the isolates, an API20 NE commercial biochemical identification system for Gram-negative non-Enterobacteriaceae (BioMèrieux, Marcy-l’Étoile, France) was used, according to the manufacturer’s instructions. The results of the API tests were interpreted using the manufacturer’s identification table (BioMèrieux, Marcy-l’Étoile, France). For DNA-based, species-level identification, single colonies were sampled with sterile inoculating loops, suspended in sterile, ultrapure water. The DNA extraction was performed with the supernatant, after incubation in a water bath at 95 °C for 20 min, followed by sonication at 50 kHz for 15 min using an Elmasonic P30H (Elma, Singen, Germany), and centrifugation at 15,000× *g* for 5 min. The samples were stored at −20 °C until further use. Oligonucleotides, rpoD70Fs and rpoD70Rs, amplifying the RNA polymerase gene sigma 70 subunit (rpoD70) of Gram-negative Gammaproteobacteria [22], were used for PCR amplification. The applied touchdown PCR assay followed the methods previously described by Yamamoto et al. [22]. The PCR products were purified with a MEGAquick-spin Plus Total Fragment DNA Purification Kit (INtRON, Seongnam-si, Korea), sequenced using a BigDye Terminator v3.1 Cycle Sequencing Kit (ThermoFisher Scientific, Waltham, MA, USA), and then searched for identities with BLASTn. 

### 2.4. Cells and Virus Isolates

EPC (Epithelioma Papulosum Cyprini, ATCC CRL-2872) cells were cultured in MEM medium (Biosera, Nuaillé, France), supplemented with 10% fetal bovine serum (FBS) (Biosera, Nuaillé, France), 1% HEPES buffer (1M), and 1% penicillin–streptomycin (Biosera, Nuaillé, France) at 25 °C.

The ECV (14612/2012) strain isolated from the brown bullhead in Hungary [13] was propagated in the EPC cell line in T175 cell culture flasks at 25 °C. Cells were infected at 80% confluency. When the complete destruction was observed, the cell culture supernatant was collected, frozen at −80 °C, then thawed, and centrifuged at 3000× *g* for 10 min to remove cell debris. Titration was executed with 10-fold dilutions of the viral inoculum in a 96-well microplate. The tissue culture infectious dose (TCID_50_/mL) was determined by the method of Reed and Muench [23].

### 2.5. Virus Challenge

Prior to the experiments, the fish were acclimated to the 21 ± 0.5 °C water temperature from 24–26 °C at the ratio of 1 °C/day. The infection of the fish was carried out by immersion at 21 °C for 1 h. The final virus concentrations in the immersion bath were 10^5^ and 10^6^ TCID_50_/mL, respectively. For all experiments, the negative control fish were handled the same way, but the MEM did not contain ECV. In the course of the experiments, the fish were kept in 50 L or 300 L tanks with aeration and water filtration at 21 ± 0.5 °C, without water recirculation or flow-through until 30 days post-infection (dpi). One-third of the water of each tank was changed with dechlorinated tap water every day. The moribund and dead fish were documented and removed daily from the tanks. Clinical symptoms were recorded, and dead fish were frozen at −20 °C until molecular examination. At the end of the experiments, all the alive individuals were euthanized by the anesthetic, clove oil [24], and liver [13] samples were collected for DNA extraction and PCR. The following experiments were conducted:

Experiment (A): Eight-week-old wels catfish were divided into 3 groups; *n* = 25 per group in 50 L tanks, with a mean individual body weight of ca. 3 g. Two groups were infected with 10^5^ and 10^6^ TCID_50_/mL ECV, respectively. The third group was used as a negative control. 

Experiment (B): The same setup as for Experiment (A) was repeated with ten-week-old fish (*n* = 25 per group in 50 L tanks, with a mean body weight of ca. 8 g). 

Experiment (C): Sixteen-week-old fish were separated into two groups (*n* = 26 per group in 300 L tanks, with a mean body weight of ca. 55 g). Due to capacity shortage, we could only carry out this experiment with two groups. One of them was infected with 10^5^ TCID_50_/mL and the other group was used as a negative control. 

### 2.6. Virus Re-Isolation

The livers were homogenized in 1 × TE buffer (pH 8.0) using a TissueLyser LT disruption instrument (Qiagen, Hilden, Germany), at 50 Hz for 5 min. Virus re-isolation was performed in T-25 flasks over monolayers of EPC cells. After centrifugation (3000× *g* for 5 min), the supernatant of the tissue samples was used at a dilution of 1:100. The cells were incubated with the supernatant at 25 °C for 1 h. The growth medium containing 10% FBS was replaced with MEM medium with 2% FBS after the incubation, and the monolayer was checked for the appearance of a cytopathic effect (CPE) daily. Isolated viruses were tested with PCR and Sanger DNA sequencing. The first few fish that died in Experiment (A) were processed immediately; the others were frozen and stored at −20 °C until the end of the third experiment, when the majority of the DNA extraction started. 

### 2.7. DNA Extraction

Samples of liver from all the individuals were homogenized using a TissueLyser LT (Qiagen, Hilden, Germany), as described above, and DNA extraction was performed with a NucleoSpin DNA RapidLyser (Macherey-Nagel, Dueren, Germany), according to the instruction manual. The DNA concentrations of the samples were measured by a Qubit 3 Fluorometer (Thermo Fisher Scientific, Waltham, MA, USA). The extracted DNA was stored at −20 °C.

### 2.8. PCR, qPCR, and Sequence Analysis

For the presence of the viral DNA, a PCR assay was applied using primers specific for the major capsid protein (MCP) gene of RVs [21]. For all the reactions, the mixture contained 25 µL DreamTaq HotStart Green PCR Master Mix (Thermo Fisher Scientific, Waltham, MA, USA), 1 µL MCPfo primer (10 pmol/µL), 1 µL MCPre primer (10 pmol/µL), 21 µL Nuclease-free water, and 2 µL DNA template (undetermined concentration). The thermal cycler condition was performed as follows: 1 cycle at 94 °C for 5 min, followed by 30 cycles at 94 °C for 30 s, 50 °C for 1 min, 72 °C for 1 min, with a final elongation at 72 °C for 10 min. After agarose gel electrophoresis, PCR products of the expected size (~500 bp) were excised from 1% agarose gels and were purified by NucleoSpin Gel and PCR Clean-up (Macherey-Nagel, Dueren, Germany). The samples were sequenced using a BigDye Terminator v3.1 Cycle Sequencing Kit (Thermo Fisher Scientific, Waltham, MA, USA), and the detection was carried out on an ABI Prism 3100 Genetic Analyzer (Thermo Fisher Scientific, Waltham, MA, USA) by a commercial service provider. The DNA sequences were analyzed with BioEdit [25] and Geneious 11.1.5 (Biomatters Ltd., Auckland, New Zealand) software packages, and the sequence identity was determined using a BLASTn algorithm at the NCBI portal.

Quantitative PCR (qPCR) was performed on the PCR positive samples of the 10^5^ TCID_50_/mL virus-infected groups. For all reactions, the qPCR mixture contained 10 µL SensiFast SYBR Hi-ROX Mix 2× (Meridian Bioscience, Cincinnati, OH, USA), 0.8 µL MCPfo (GTT CAT GAT GCG GAT AAT GTT GT) primer (10 pmol/µL), 0.8 µL MCPre (ACC TCT ACT CTT ATG CCC TCA GC) primer (10 pmol/µL), 5.4 µL Nuclease-free water, and 100 ng DNA template (3 µL). The thermal cycler condition was performed as follows: an initial denaturation step at 95 °C for 2 min, followed by 40 cycles of 95 °C for 5 s and 65 °C for 30 s.

### 2.9. Statistical Analysis

Statistical analysis was performed for Experiments (A), (B), and (C) using R Commander (version i386 4.1.1.) (Vienna, Austria) software. Cumulative mortality data were statistically analyzed with Fisher’s exact test, and graphs were generated with Microsoft Excel (2016; version 16.0.5278.1000). The 10^5^ TCID_50_/mL virus-infected groups were compared in all the experiments, to examine the ECV mortality of fish at different ages and to investigate the age-related mortality. Cq values of qPCRs (i.e., the PCR cycle number at which the sample’s reaction curve intersects the threshold line) were analyzed with one-way ANOVA, followed by a Tukey post hoc test. The difference was considered significant at *p*-value less than 0.05. 

## 3. Results

### 3.1. Virological, Bacteriological, and Parasitological Examinations

During the acclimatization period, no clinical signs of any diseases were detected. Pre-experiment screening for ranavirus DNA gave negative results in all groups and light microscopy examinations could not detect any parasites. However, in Experiment (A), at 8 days post-infection (dpi), trophonts of the white spot protozoan, *Ichthyophthirius multifiliis* (‘ich’), were detected in all three groups. FMC treatment was immediately started, and after four days, the parasite infection vanished in all groups. During this period, none of the control fish died. For the upcoming experiments (Exp. B and C), we treated the fish with FMC prior to the experiments.

In Experiment (C), sporadic mortalities were observed, both in the infected and in the control groups. The bacteriological examination of the spleen and kidney of the fish revealed the presence of *Aeromonas veronii*, a Gram-negative, facultative pathogen bacterium that might be responsible for the deteriorating health status of fish.

### 3.2. Virus Challenge

**Figure 1:**
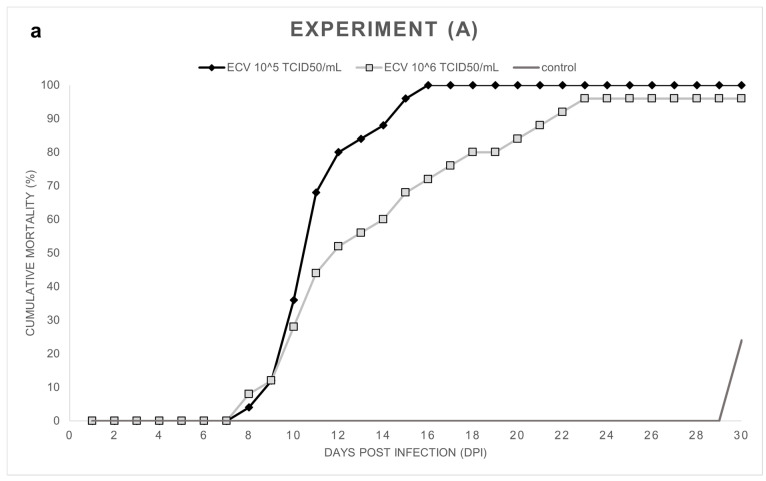

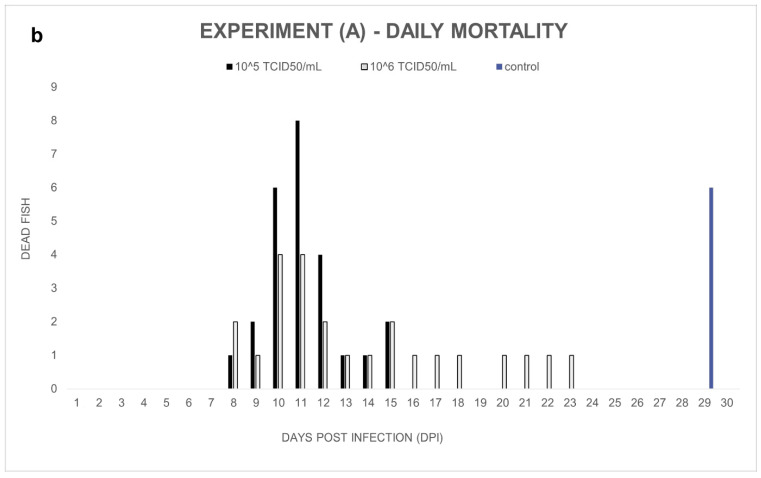
(**a**) Cumulative mortality in Exp. (A), wels catfish (*Silurus glanis*) (*n* = 25/group) were infected at 8 weeks of age by bath with ECV at 21 ± 0.5 °C for 1 h. Mortality was recorded from 0 to 30 dpi, including the control group. The number of the control group was reduced due to cannibalism. (**b**) Daily mortality in Exp. (A) shows the number of dead fish per day.

Experiment (A): Notable cumulative mortality was recorded in the groups infected by ECV: 100% in the group exposed to 10^5^ TCID_50_/mL ECV, and 96% in the group exposed to 10^6^ TCID_50_/mL virus (Figure 1a). The statistical analysis confirmed that there was no significant difference (*p* = 1.0000) between the level of mortality caused by the two virus concentrations. The decease started at 8 dpi and stopped at 23 dpi (Figure 1b). Necropsy was performed on every fish, and several macroscopic lesions, including anemia and whitish lesions, mainly in the liver, were detected. On the skin near the lower jaw or the gill, and in the fins of fish, diffuse subcutaneous hemorrhages could be detected. Anorexia was observed in all the virus-infected groups (Exp. A, B, and C). In the control group, cannibalism caused 24% mortality in the last two days of the experiment. Still, significant differences (*p* < 0.0001) were detected between the mortality rates of the control and the infected groups.

**Figure 2:**
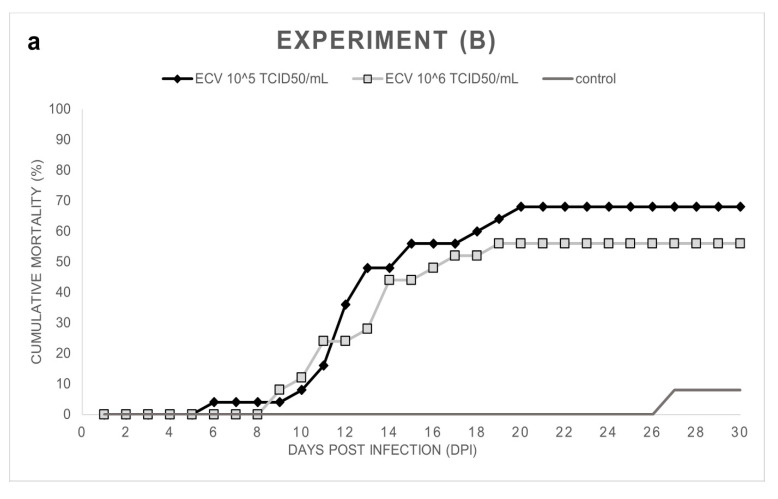

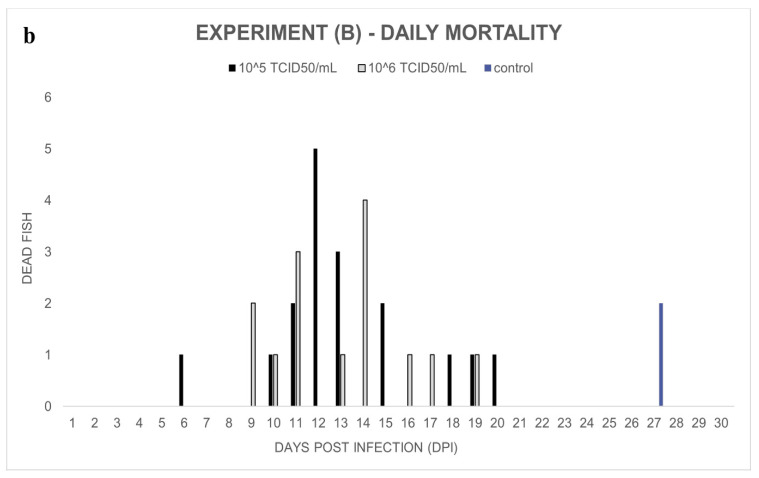
(**a**) Cumulative mortality in Exp. (B), wels catfish (*Silurus glanis*) (*n* = 25/group) were infected at 10 weeks of age by bath with ECV at 21 ± 0.5 °C for 1 h. Mortality was recorded from 0 to 30 dpi, including the control group. (**b**) Daily mortality in Exp. (B) shows the number of dead fish per day.

Experiment (B): In the ECV-infected groups, 68% (10^5^ TCID_50_/mL) and 56% (10^6^ TCID_50_/mL) cumulative mortalities were observed between day 9 and day 20 post-infection. In the control group, two wels catfish died on day 27 (Figure 2a,b). Significant differences (*p* < 0.0001 and *p* = 0.0003) were detected among the mortality rates of the control and infected groups. However, there was no significant difference (*p* = 0.5607) between the percent mortality caused by the two virus concentrations.

**Figure 3:**
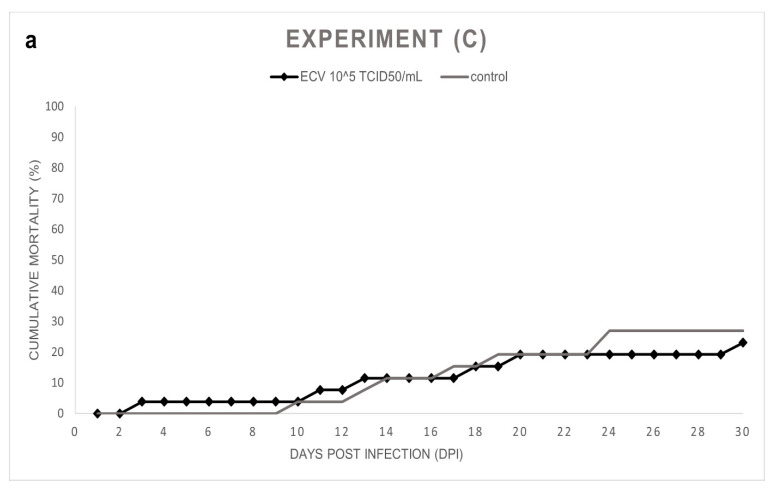

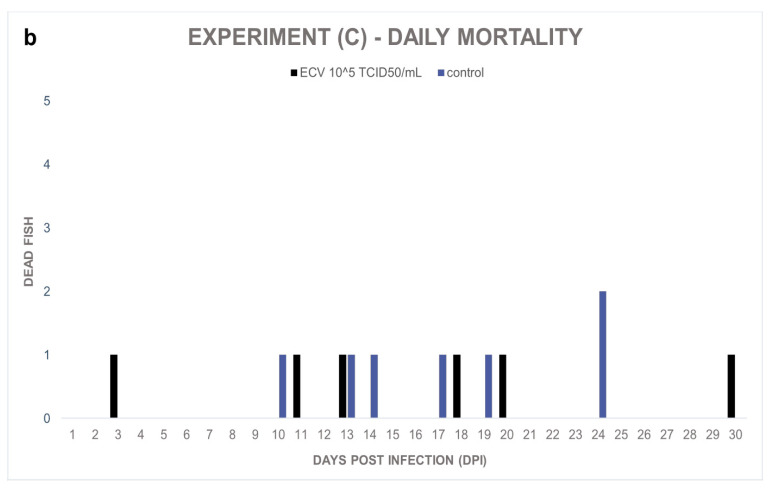
(**a**) Cumulative mortality in Exp. (C), wels catfish (*Silurus glanis*) of 4 months (*n* = 26/group) were infected by bath with ECV at 21 ± 0.5 °C for 1 h. Mortality was recorded from 0 to 30 dpi, including the control group. (**b**) Daily mortality in Exp. (C) shows the number of dead fish per day.

Experiment (C): In the course of the experiment, the sixteen-week-old control and the infected fish showed a nonsignificant (*p* = 1.0000) but similar level of sporadic mortalities, 27% and 23%, respectively (Figure 3a,b). In the infected group, anorexia and subcutaneous hemorrhages were also observed in the surviving fish. In the control group, the presence of the virus was not detected, and the external signs of virus infection were not observed. However, the behavior of these wels catfish was aggressive; wounds were found on the skin of multiple individuals.

### 3.3. Virus Re-Isolation

**Figure 4:**
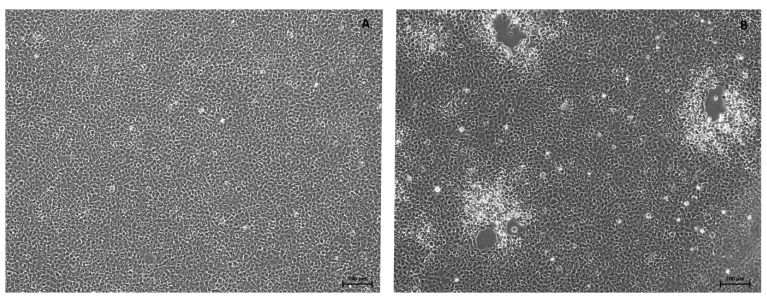
The first three dead wels catfish from Experiment (A) were dissected immediately for virus re-isolation. The ECV was successfully isolated from all of them. (**A**): the control EPC cells and (**B**): the CPE caused by the ECV 48 h after the inoculation with the sample originating from the first dead fish.

At day 2 post-infection, a definite CPE was observed on ECP cell monolayers inoculated with the homogenized samples of the first three dead and promptly dissected wels catfish from Experiment (A) (Figure 4). Two days later, the CPE was complete. Subsequently, the PCR and the sequence analysis confirmed that the ECV used for experimental fish infection was isolated. Unfortunately, isolation attempts from all of the frozen samples failed. 

### 3.4. PCR, Sequencing, and qPCR

In all the experiments, none of the control fish produced positive results for ECV, and all the dead fish from the infected groups proved to be positive for the presence of ECV (Table 1). In Experiments (A) and (B), the surviving fish from the infected groups were ECV-negative with PCR. In contrast, in Experiment (C), 11 samples from the 20 surviving fish gave positive results (Table 1). A total of 10% of the positive samples were selected randomly for DNA sequencing and analyzing. All of them shared 100% nucleotide sequence identity with the MCP gene of the ECV published previously (GenBank Acc. No.:FJ358608).

The mean and standard deviation of the Cq values were calculated in the groups exposed to 10^5^ TCID_50_/mL ECV in all three experiments: the mean Cq was 19.57 (8.59) in Experiment (A); 13.54 (4.19) in Experiment (B), and 30.76 (8.24) in Experiment (C). 

### 3.5. Statistical Analysis

The overall cumulative mortalities of the virus-infected groups at the 10^5^ TCID_50_/mL dosage were compared in all the experiments, and a significant difference (*p* < 0.0001) was found among the three virus-infected groups.

The real-time quantification results were compared, and a significant difference (*p* < 0.0001) was found in the estimated amount of ECV DNA among the three age groups. The pairwise comparisons of the means showed significant differences in all cases: *p* = 0.0393 between the 8- and the 10-week-old groups. The 16-week-old group compared with the other age groups resulted in a *p* < 0.001 value. 

## 4. Discussion

A study published previously investigated the susceptibility of wels catfish to different RV isolates (ECV, ESV, EHNV, Frog virus 3, Rana esculenta virus, Bohle iridovirus, guppy virus 6, short-finned eel virus, and doctor fish virus) and found that only the ESV and ECV might cause notable disease in fish [20]. Here, we intended to examine if the ECV from the brown bullhead isolated in Hungary [13] may cause disease in wels catfish, whose importance is growing rapidly in the aquaculture of our country. We also wanted to know whether the severity of the ECV infection was age-related in wels catfish. To answer our questions, three experimental challenges with ECV were carried out. Although the optimal temperature range for ranavirus replication is 20–28 °C [26], the previously published experimental infections were executed at 15 °C and 25 °C [20]. We applied an intermediate temperature, imitating the average temperature during the reproduction period of wels catfish in Hungarian water sources.

Significant mortality caused by ECV was recorded in Experiments (A) and (B) compared to the control groups. All the dead fish from the ECV-infected groups were PCR positive, but all the fish were PCR negative in the control groups. The external and internal signs observed in the diseased fish were consistent with those of the natural infections detected previously [13]. The virus re-isolation from freshly dissected fish was successful, in accordance with the PCR results, but it failed from frozen samples. The sudden ‘ich’ infection might have influenced the results of Experiment (A), but it did not lead to mortality in the control group. In the last days, only cannibalism caused losses, but by then, the mortality in the infected groups had already stopped. Unexpected events occurred in Experiment (B) in the control group; two wels catfish died in the last days when deaths had ceased in the infected groups by that time. 

Interestingly, the percent mortality was higher in both experiments ((A) and (B)) in the groups challenged to lower virus doses; however, these differences were not significant. Trials on a larger population would be needed to confirm the real differences between the two doses applied.

The cumulative mortality was significantly lower in the infected groups of Experiment (C) than in the previous experiments, and similar percent mortality was also observed in the control group. The daily mortality in Experiments (A) and (B) had peaks at 11–12 dpi. This is a typical distribution pattern for virus-caused mortalities [27]; however, it was not observable in Experiment (C). In the latter case, *Aeromonas veronii* infection might also have contributed to the mortality (in both the exposed and the control groups). Furthermore, in the control group, numerous wounds were found in the skin of the fish compared to the ones in the exposed group, which could be the result of the more aggressive behavior due to the lack of a negative health impact (i.e., anorexia), which was observable in all the exposed groups. These injuries and wounds could have also played a role in the unexpected mortalities. The viral loads measured by qPCR were significantly different in the three age groups. Interestingly, the highest viral load was observed in the exposed individuals of Experiment (B), although the mortality dropped from 100% to 68% in this group. The viral load was remarkably lower in Experiment (C) (3–5 orders of magnitude lower than in Exp. (A) and (B)). This finding might imply that the immune system of the wels catfish has become more developed at this age and thus it was able to cope with viral infection.

According to the PCR results, all the surviving fish in the exposed groups in Experiments (A) and (B) were negative, in contrast with Experiment (C), in which 11 of the 20 survivors proved to be positive for the presence of viral DNA, even after 30 dpi. Our finding might imply that ECV could induce persistent infection in older wels catfish. Although PCR positivity does not confirm the ability of virus reproduction, it is possible that older fish transmit the infection to the younger ones, even transmitting fatal diseases in intensive fish farms [13,18]. Unfortunately, testing this hypothesis under experimental conditions would be difficult due to the aggressive behavior and cannibalism of the wels catfish. 

Formerly, the susceptibility of wels catfish to isolates from ECV from France [10] and ECV-24 from Italy [12], with a 10^4^ TCID_50_/mL concentration at 15 °C and 25 °C, was investigated. The ECV isolate from France has shown particularly low, 8% mortality in 10 g wels catfish at 15 °C. At 25 °C, this ECV strain induced 55% mortality, whereas the ECV-24 isolate from Italy caused 81% mortality of 0.5 g fish (age data have not been published in this study) [20]. The high mortality rate of juvenile wels catfish caused by the Hungarian isolate is similar to that of the one from Italy, although we used a higher (10^5^ TCID_50_/mL) dose for the virus challenges, the water temperature was lower, and the fingerlings were older in our experiments. Hence, both the Hungarian and the Italian isolates seem to pose a high risk for juvenile wels catfish in aquaculture. 

Unfortunately, the ECV-24 infection in older fish was not studied; however, the authors suspected the importance of the age of the fish as a predisposing factor besides the water temperature [20]. In our study, we found that juvenile wels catfish display age-related mortality to ECV infection at 21 °C. It seems that wels catfish younger than 4 months of age are highly susceptible to ECV; therefore, it would be worth considering developing protective measures against the virus infection.

## 5. Conclusions

Our study proved that the ECV isolated from the brown bullhead in Hungary could cause severe losses among juvenile wels catfish populations. The mortality rates induced by the virus seem to be age-related. The wels catfish seems to be most vulnerable to ECV infection in its first three months of life; thus, the importance of the prevention of virus infection in juvenile wels catfish in intensive fish farms is unquestionable. Furthermore, our results suggested that the ECV could cause persistent infection in older wels catfish and hence they may be considered as carriers of the virus. 

## Figures and Tables

**Table 1:** Percent cumulative mortality in Experiments (A), (B), and (C) at the two virus dosages used, and the number of positive PCR results from the dead or the surviving fish.

Experiments	Weight	Group	Mortality		Positive PCR Results/Total Tested Fish	
			No. Dead Fish/Total in Group	Percent Mortality (%)	Dead Fish	Surviving Fish
**Experiment (A)**	ca. 3 g	10^5^ TCID_50_/mL dose	25/25	100%	25/25	0/0
		10^6^ TCID_50_/mL dose	24/25	96%	24/24	0/1
		control	6/25	24%	0/6	0/19
**Experiment (B)**	ca. 8 g	10^5^ TCID_50_/mL dose	17/25	68%	17/17	0/8
		10^6^ TCID_50_/mL dose	14/25	56%	14/14	0/11
		control	2/25	8%	0/2	0/23
**Experiment (C)**	ca. 55 g	10^5^ TCID_50_/mL dose	6/26	23%	6/6	11/20
		control	7/26	27%	0/7	0/19

## Data Availability

Not applicable.
